# A Review of Federated Learning in Agriculture

**DOI:** 10.3390/s23239566

**Published:** 2023-12-02

**Authors:** Krista Rizman Žalik, Mitja Žalik

**Affiliations:** 1 Faculty of Electrical Engineering and Computer Science, University of Maribor, 2000 Maribor, Slovenia; mitja.zalik@um.si; 2 Faculty of Natural Sciences and Mathematics, University of Maribor, 2000 Maribor, Slovenia

**Keywords:** federated learning, agriculture, architecture, data partitioning, federation scale, aggregation algorithms, communication bottleneck

## Abstract

Federated learning (FL), with the aim of training machine learning models using data and computational resources on edge devices without sharing raw local data, is essential for improving agricultural management and smart agriculture. This study is a review of FL applications that address various agricultural problems. We compare the types of data partitioning and types of FL (horizontal partitioning and horizontal FL, vertical partitioning and vertical FL, and hybrid partitioning and transfer FL), architectures (centralized and decentralized), levels of federation (cross-device and cross-silo), and the use of aggregation algorithms in different reviewed approaches and applications of FL in agriculture. We also briefly review how the communication challenge is solved by different approaches. This work is useful for gaining an overview of the FL techniques used in agriculture and the progress made in this field.

## 1. Introduction

The influence of smart sensors in agricultural development is based on recent technological improvements. The use of spatial, temporal, and image data can enhance prediction accuracy in agriculture. Sensors that capture data related to crops, weather, soil, and other agricultural variables at diverse intervals, improve the efficiency of machine learning (ML), improve agricultural management, increase productivity, and make agriculture more efficient and sustainable.

### 1.1. Machine Learning (ML)

Machine learning (ML) uses data and algorithms to learn with, gradually improving its accuracy [1,2].

Applying machine learning in agriculture allows for more efficient and precise farming since machine learning can help establish knowledge-based farming systems [3]. ML algorithms can analyze large datasets of agricultural data, such as crop yields, soil, and weather data, to identify patterns and make knowledge of agricultural data. This knowledge is used by knowledge-based farming systems that can help farmers to make better actions.

Machine learning offers new opportunities to predict and understand data and processes in different agricultural areas. In [4], the authors analyzed the use of ML for different agricultural areas: water management, soil management, livestock production and management, and crop management (including yield prediction, disease detection, weed detection, and species recognition). 

### 1.2. Deep Learning (DL)

Deep learning (DL) is a subset of machine learning (ML). DL extends classical ML by incorporating depth and complexity into models, which enables learning through multiple levels of abstraction [5,6]. DL consists of multiple processing layers that enables hierarchical learning [7]. The DL hierarchy allows learning in a non-linear approach, with each layer integrating additional information in a hierarchical way.

Many different deep learning architecture models have been proposed, including deep neural networks (DNNs), convolutional neural networks (CNNs), recurrent neural networks (RNNs), and others (see Table 1), which have already been successfully applied to various fields, including agriculture. 

Convolution neural networks (CNNs) are very effective for tasks like object detection and image classification [8]. The hidden layers in CNNs consist of a series of convolutional layers, which extract high-level characteristics from the input. Multiple fully connected layers follow the convolutional ones.

Recurrent neural networks (RNNs) [9] model temporal dynamic behavior. They contain loops, and connections between nodes create cycles, allowing the outputs from some nodes to affect subsequent inputs to the same nodes.

A comparison of the classification and regression performance of deep learning in agriculture with other existing popular techniques is presented in [10]. Their findings prove that deep learning significantly improves the performance of classification and prediction problems, providing high accuracy. They identified sixteen agricultural applications of deep learning in surveyed research works, including weed identification, land cover classification, plant recognition, fruit counting, and crop type classification. The surveyed works demonstrated improvements in the performance of prediction problems.

### 1.3. Edge Computing

With the rapid development of the Internet of Everything, the number of smart devices connected to the Internet is increasing, producing large-scale data, which causes slow response speed, poor security, and poor privacy [11]. A new paradigm has been developed to make computing closer to the source of the data at the edge of the network [12]. Edge computing improves response time, saves bandwidth, reduces costs, enhances speed, and improves security. 

Edge computing is a distributed information technology (IT) architecture in which client data are processed at the network’s periphery, as close to the source data as possible [13]. Data are not transmitted to a central data center for processing and analysis; instead, processing occurs where the data are generated. Edge computing aims to decentralize computing processes and resources, placing them closer to data sources, which improves privacy and security concerns. It also reduces costs efficiently by processing data on edge devices, eliminating the need to transfer them to a central server and allocate extensive storage [13]. 

Deploying deep learning (DL) services through edge computing has increased since DL can be seamlessly integrated into edge computing frameworks, creating intelligent edges for dynamic edge maintenance and management [14].

### 1.4. Federated Learning (FL)

Many machine and deep learning methods are centralized machine learning methods using only data stored in a centralized data repository. They do not ensure data privacy and require large datasets during the training process.

However, many resources used in machine learning in agriculture, such as crop management, soil, or weather data, are distributed. They reside on smart farming devices or are stored on servers owned by different organizations. Sufficient data for training DL algorithms in agriculture are generated from sources like weather data, high-resolution drone images, satellite data, and other spatial and temporal data. Agriculture generates significant real-time data through low-cost and low-energy-consuming sensors and devices [15]. Ground surveys in agriculture are expensive and typically cover only small areas or a selection of sample farms. In contrast, remote sensing offers cost-effective monitoring in agriculture [16]. These devices generate real-time data and rely on open internet communication. This requires minimizing the risks associated with security and data privacy violations. Data privacy is also crucial for farmers, not only for large organizations [17].

One solution to the data security problem involves cooperation among multiple data owners to train and use shared machine learning models while preserving all training data locally. This is achievable with an emerging machine learning technology called federated learning (FL). The start of FL was introduced by the Google team in October 2016 [18], and the first aggregation algorithm, FedAvg, was introduced in 2016 [19]. FL is a type of machine learning in which different clients or users collaborate to train a model while maintaining decentralized control of their data [20]. It reduces the risk of privacy violations [21]. FL trains machine learning models across multiple decentralized clients using only local data samples, avoiding the need to exchange data with other clients or a central server [21].

FL [22] is increasingly being adopted across various application domains as a novel technology for data science on distributed decentralized data (i.e., data that are not exchanged or shared but remain with their owners). Due to privacy concerns and the effectiveness of deep learning, machine learning methods are transitioning towards FL techniques. FL involves leaving the training data distributed on mobile devices or other clients while learning a shared model by aggregating updates to locally computed parameters of locally trained machine learning networks. Clients do not exchange data; instead, they exchange parameters of their models, such as weights and biases of deep learning networks. In FL, multiple distributed clients train their own machine learning model using their local data. These clients then send their local models to a central server, which merges the models and orchestrates the learning process. New clients can join the FL environment, but this requires additional learning iterations.

First, a randomized, global model for a neural network, such as a convolutional neural network, is generated at the server site. The FL training iteration starts with this global model on the central server and proceeds through the following steps.

- A subset of candidate clients is selected for the FL iteration;
- The global model is sent to the selected client edge devices;
- Each client learns its model using only its local data and computes a local update on the model, typically using Gradient Descent;
- The central server collects local model updates from edge devices and computes an aggregated model update. This aggregation step may involve lossy compression to enhance communication efficiency [20,23];
- The central server uses the computed aggregated update to update the global model;
- Subsequently, the server returns the global model parameters to the clients for the next iteration of model learning.

FL differs from centralized learning (CL) in the following features. In CL, data are collected on the server, while in FL, they are collected on the clients, where they are used for distributed learning. Training is performed on the server in CL and on edge devices in FL. No aggregation is necessary in CL, while in FL, aggregation is performed on the server. In FL, model updates are shared, while in CL, local data are shared and collected on the server. In CL, data are submitted to the server once, whereas in FL, model updates are iteratively transferred to the server.

FL is an efficient technology, particularly when training on real data from distributed devices. It offers advantages over training on centralized data located in one central place or when data are sensitive. Additionally, the computational power is distributed amongst clients, which requires more resources for learning about clients within the federated network. 

FL has numerous applications [24], and it holds significant importance in agriculture. There is still no systematic review of the use of FL in applications in agriculture. 

This study provides a comprehensive overview of various FL solutions and their applications in agriculture. We classify these applications based on different agricultural areas, FL architectures, federation levels, data distribution, and the aggregation algorithms used to generate the global model. This review aims to enhance the understanding of FL technology and to promote its use in agriculture.

The contributions of this paper are as follows. We discuss FL technology and classify FL applications in agriculture based on data partitioning methods, architecture, scale of federation, and aggregation algorithms for generating the global model. We provide an overview of the technological features of FL in agriculture. We discuss the challenges and opportunities for FL technology and its applications in agriculture, focusing on resolving communication bottlenecks.

The rest of this paper has the following structure. Section 2 describes materials. Section 3 provides a summary of the methods used for this overview. Section 4 summarizes the FL paradigm, surveys the aggregation algorithms used for global model generation, classifies FL approaches, and reviews FL applications in agriculture. Section 5 discusses the challenges and opportunities of FL in agriculture. Section 6 concludes the paper.

## 2. Materials 

This overview contains relevant research works involving FL in different areas and aspects of agriculture found using several databases and search engines, including Google Scholar, Science Direct, IEEE Xplore, PubMed, ACM Digital Library, and MDPI.

Our search strategy primarily employed keywords such as ‘federated learning’ and ‘agriculture’ or ‘model compression’. Additionally, we used combinations of the keyword ‘federated learning’ with specific agriculture-related terms such as crop, water, soil, animal, and livestock. 

We used guidelines that Siddaway et al. [25] proposed to perform an effective systematic literature review for English regular papers and preprints. First, we reviewed the titles and abstracts of the articles and excluded those that did not mention FL in agriculture. Finally, the full text of the remaining articles was read, and articles that only used existing methods of FL and did not propose any novelty were excluded. As a result of this process, a final set of 11 articles was included in this survey.

## 3. Methods

We summarize FL technology and its applications in agriculture based on data partitioning, architecture, scale of federation, and aggregation algorithms for generating the global model. 

### 3.1. Data Partitioning

FL supports three types of data partitions: horizontal, vertical, and hybrid data partitioning. Data partitions determine the type of FL (Table 2), such as horizontal FL, vertical FL, and federated transfer learning [26].

#### 3.1.1. Horizontal Data Partitioning

Horizontal FL, known as sample-based FL, is a common architecture in FL systems. Horizontal FL is also called homogeneous FL because the datasets of different clients have the same features, but each client’s samples are largely non-overlapping.

This is the most common type of FL, where the data from each client are similar. Therefore, the same model can be used for all clients, making aggregating the data on the server easier. Ref. [27] states that the main benefit of horizontal FL is that it allows for independent learning across clients and improves security.

In order for the server to achieve better convergence, some algorithms like MIME [28] also send local gradients and other statics to the server.

#### 3.1.2. Vertical Data Partitioning

In vertical FL, also known as feature-based FL, data are vertically partitioned. It is used when two or more clients have the same or similar sample spaces and different feature spaces. Entity alignment techniques are used to find overlapping data samples among clients, which are then used for training. Vertical FL is also called heterogeneous FL.

The labels are either made available to the server [29] or stored on a designated client [30]. Vertical FL computes a machine learning model’s cost function and gradients by collaboratively sharing the unique features of the samples collected by different clients [31]. The global inference model is on the server, while local clients train the local models with the local features. An example of an algorithm is Vertical Asynchronous Federated Learning (VAFL) [32].

#### 3.1.3. Hybrid Data Partitioning and Transfer FL

In hybrid data partitioning, the local dataset at each sensor contains a partial sample space and a partial feature space [33] with little overlap in both the feature space and the sample space. Hybrid FL has partially overlapped feature spaces and sample spaces. Hybrid FL differs from conventional FL with horizontal data partitioning, which has similar sample spaces, and vertical data partitioning, which has similar feature spaces.

Hybrid data partitioning has the following characteristics [34]:

- Local and global model. Each client trains its local model on data with a subset of features. The server global model supports all the features. Each client has some features of all training samples, as in vertical FL.
- Limited data sharing of labels (features). In horizontal FL, the clients do not share labels; in vertical FL, labels may be made available to the server. The FL system needs to deal with both types of clients.
- Sample synchronization. In hybrid FL, like in vertical FL, not all clients have all the samples. The problem of aggregation is even greater in hybrid FL systems because not all clients have all samples, and algorithms do not require clients to synchronize their sample sets.

Many works have been developed for horizontal FL containing clients sharing the same features and largely non-overlapping samples and vertical FL containing clients sharing the same samples and largely non-overlapping features. However, the hybrid data partitioning with transfer FL remains less explored.

Transfer FL allows knowledge to be transferred across domains that do not have many overlapping features and samples. In practice, a client may contain only some subjects and some features, and no client has all the features or all the subjects. Such clients can participate only in transfer FL. Clients in transfer FL, such as trade chains, insurance companies, or banks, serve just a fraction of all customers and have only their partial data.

Many applications have been developed using horizontal FL, some using vertical FL, but applications using transfer FL remain rare. Examples of applications of transfer FL are in the healthcare domain [33], autonomous driving [35], and image steganalysis [36]. 

In [34], a new model-matching-based problem and an efficient algorithm formulation for hybrid FL is introduced, and an efficient algorithm that can collaboratively train the global and local models to deal with full and partial featured data is proposed.

### 3.2. Architecture

FL systems can have centralized and decentralized architecture [19]. These two architectures differ solely in the client–server communication, while both outcomes are the same.

#### 3.2.1. Centralized FL Architecture

**Figure 1:**
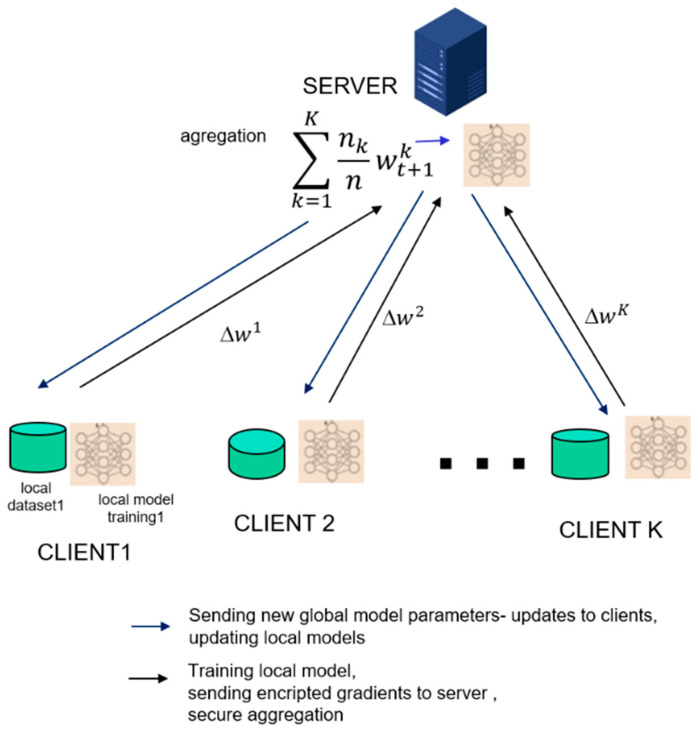
Architecture for a centralized FL system.

In the centralized architecture, a server hosts the globally separated model, and all parameter updates occur within this global model. Clients conduct local training using their own data (refer to Figure 1) and perform learning that can be synchronous and involves several steps.

- Initially, a global model is transmitted to edge devices (clients);
- Each client trains its model with its local data and sends its local model parameters to the central server for aggregation, thereby improving the global server model;
- The central server aggregates the model parameters and returns the updated global parameters to the clients;
- Local models are initialized with the received global parameters and are further trained;
- This process repeats until it reaches the maximum number of iterations or until the server model converges;

In the centralized federated architecture, *m* clients (users) collectively learn a global model without sharing their data directly with one another. Clients only exchange weights (*w*) of their local models with the server. The optimization problem is described with Equation (1) [19]:

(1) $$\underset{w\epsilon R^{d}}{\min}f\left(w\right)≅\frac{1}{m}\overset{m}{\underset{j=1}{\sum }}f_{j\;\left(w\right)\;}$$

where $f_{j\;}:R^{d}\to R\;\mathrm{represents}\;\mathrm{the}\;\mathrm{loss}\;\mathrm{function}\;\mathrm{corresponding}\;\mathrm{to}\;\mathrm{client}\;j$.

Centralized FL architecture has been the most common approach since the beginning of FL. A centralized approach has the following disadvantages: delays due to bottlenecks, potential for system errors, and reliability concerns in creating a global model.

#### 3.2.2. Decentralized FL Architecture

In the decentralized architecture, one client is randomly selected at the beginning of the epoch to perform all the server’s tasks. This client is responsible for updating the global model and communicating its parameters to other clients. Decentralized FL systems can be implemented in various technologies, such as peer-to-peer (P2P) networks, graph-based systems, and blockchain technologies.

Decentralized FL [37,38] enables direct communication between clients, and the central server is not needed, which saves communication and computational resources. The pointing and peer connections in the communication network are adaptively configured and changed according to the use case. Clients can be connected based on geographical neighbors or their similarities. 

Two paradigms of decentralized FL are aggregate and continual. In aggregate decentralized FL, the client first aggregates the models of past clients and then learns from the aggregated models continually, where the client learns directly from the model of the previous client. 

In continual decentralized FL [39], the client can obtain a more personalized model while saving computational and storage resources. In continual decentralized FL, clients do not need to wait before the local learning process for all data to be collected and aggregated, and clients always have the latest version of the model. Fewer communication, computation, and storage resources are required.

There are some problems with continual decentralized FL. The client iteration order has a significant impact on the model performance. The previous knowledge can be forgotten without an appropriate learning rate and training epoch in learning the current client’s knowledge. 

#### 3.2.3. FL Architecture and Data Partitioning

Depending on the organization of data (features and samples) among nodes, decentralized FL architecture can be divided into the same three types as centralized FL: horizontal FL [40], vertical FL [41], and transfer FL [42].

Horizontal FL is the most used method in decentralized FL with performing sample federation. It is used when there are many overlapping features and few overlapping nodes, typically in cross-device FL systems. Vertical FL and transfer FL are more complex to use. Vertical FL is used when there are many overlapping nodes and few overlapping features, and transfer FL is used when there is a limited feature and sample intersection between nodes.

### 3.3. Aggregation Algorithms

In the FL system, the server receives weights from trained local machine learning models and performs mathematical merging operations on the weights to create the shared global model $w_{t}$, where *t* represents the epoch number. The server can employ various mathematical operations for weight merging. 

In Federated Averaging (FedAvg) [19], aggregation is implemented using an averaging function. It averages the weights of different local models to generate new weights. FedAvg relies on the following parameters: the fraction of clients *C* selected for training, the local mini-batch size *B*, and the number of local epochs *E*, which indicates the total number of iterations of learning performed on the local data before updating the global model.

- Firstly, FedAvg initializes the global model $w_{0}\;$ randomly;
- FedAvg selects a subset of clients, denoted as $C_{t}$, |$C_{t}$| = C K, with C and K being parameters, both greater than or equal to 1, at each iteration;
- Next, it sends the current global model $w_{t}$ to all clients in subset $C_{t}$ (see Figure 1);
- The local models on each client *k* are updated to the shared model, $w_{t}^{k}$ ← $w_{t}$;
- Each client partitions their local data into batches of size *B* and perform epochs of Stochastic Gradient Descent (SGD);
- After training, each client sends its updated local model, $w_{t+1}^{k},$ to the server;
- The server computes a weighted sum of all received local models to obtain the new global model, $w_{t+1}$.

In FedPer [29], the model is split into base and personalized layers. For the two-layered CNN, the last dense layer is the personalized layer; the others are base layers. Personalized layers are not sent to the server because only the federated server aggregates the base layers. 

A communication-efficient FedAvg, known as CE-FedAvg, which reduces the convergence time compared to FedAvg, has been proposed in [43]. CE-FedAvg decreases the required rounds to achieve the desired accuracy and reduces the total amount of data downloaded per round compared to FedAvg.

Stochastic Gradient Descent (SGD) has shown great results in deep learning. SGD can be applied directly to the federated optimization problem, where a single batch gradient is performed per round of communication. FedSGD [44] uses only different random subsets of clients for each learning round, converging faster in each round. It requires many rounds of training to produce good models. 

FedProx is an improved version of FedAvg for considering heterogeneity in FL [45]. FedProx considers variations in computing power and different factors in devices participating in FL training. FedProx also introduces a proximal term to handle inconsistencies in local updates. The results of the experiment indicate that FedProx can achieve good results in heterogeneous settings.

The FedMA algorithm is used to build a shared model to aggregate CNN and LSTM models in FL [46]. FedMA does not simply average the weights of neurons in a layer but uses approach that considers the feature extraction signatures of neurons. This allows FedMA to merge similar neurons and produce a more compact and efficient model. The experimental results show that FedMA works well on heterogeneous clients, surpassing FedAvg and FedProx in several training rounds. FedMA is a layer-wise learning scheme that incorporates matching and merging nodes with similar weights. 

P-FedAvg extends the well-known FedAvg algorithm by allowing multiple parameter servers to cooperate and train a learning model together [47].

In [48], a new architecture named EdgeFed is proposed. Edge devices and servers complete local updates, and global aggregation is executed between edge servers and the central server.

### 3.4. Scale of Federation

Regarding the scale of training models and number of clients, FL can be classified into cross-device FL and cross-silo FL [29].

Cross-device FL has many edges (i.e., IoT and mobile phones). Each client has a unique local row dataset for training local learning models.

There are also FL applications with only a few edge devices, which form cross-silo FL [49]. The number of clients is small, usually below a hundred. Clients in cross-silo FL are organizations or companies. Data from each organization in data silos are always available and usually have different example features. 

#### 3.4.1. Scale of Federation and Data Partitioning

In the cross-device FL systems, the data are assumed to be partitioned by samples, and horizontal FL is usually performed.

Horizontal partitioning by samples also occurs in cross-silo FL systems when a single company cannot centralize its data or when different companies with similar objectives participate in FL to improve their models. 

In the cross-silo FL systems, in addition to horizontal partitioning by samples, vertical partitioning by features occurs when two companies in different businesses have the same or very overlapping set of customers. This vertical partitioning often does not involve a central server and has continual decentralized FL architecture. 

Cross-silo FL can be used when two companies in different businesses do not have very overlapping customer sets [20].

#### 3.4.2. Scale of Federation and Architecture

The decentralized FL architecture and the centralized FL architecture occur in both types of federation cross-silo and cross-device . 

Cross-silo centralized FL and decentralized FL systems consist of nodes, which are organizations or data centers [50] with large amounts of data. 

Cross-device decentralized FL systems usually have a relatively large number of nodes, which are on-edge devices or robots like UAVs with weak communication between nodes if they are not in a close coverage radius [44] and with a small amount of data (about thousands of samples) and limited computational power [51]. 

## 4. Results

### 4.1. Use Cases of FL Applications in Agriculture 

In agriculture, farmers often have sensitive data that they do not want to share with others. FL is ideal for agricultural applications because FL is a machine learning technique that allows multiple devices to train a shared model without sharing their data. 

In this study, we classify FL applications in agriculture according to architecture, data partitioning methods, scale of federation, and aggregation algorithms for generating the global model. 

All 11 considered FL applications in agriculture, summarized in Table 3, have a centralized architecture. The scale of the federation is for all discussed FL applications except one cross-device. We discuss the various frameworks used in FL in different agriculture areas and different trained models with machine learning and deep learning used in FL applications in agriculture. 

Manoj et al. [52] used FL to train the prediction model of yield on a horizontally distributed dataset located on different client devices. The FedAvg algorithm is used to train deep regression models like ResNet-16 and ResNet-28 to prove the effectiveness of decentralized learning of agricultural data.

Kumar et al. [53] proposed PEFL, a deep privacy-encoding-based FL framework that uses a perturbation-based encoding and long short-term memory-autoencoder for avoiding data privacy violation and intrusion detection. They used an FL-based gated recurrent unit neural network algorithm (FedGRU) for intrusion detection from the encoded data.

Durrat et al. [54] developed a machine learning model that shares data across supply chains.

Atico et al. [55] evaluated the performance of FL system with five CNNs trained in a distributed environment and measured their training time compared to their classification performance. FL was efficient in predicting crop leaf diseases from images. The authors confirmed that training time is inversely proportional to accuracy. They also proved a correlation between the number of CNN parameters in FL and the volume of data exchanged during training. 

Mao et al. [56] proposed a framework named the Federated Learning Framework for Animal Activity Recognition (FedAAR) for automated animal activity recognition using a distributed model across multiple farms without sharing personal data.

Khan et al. [57] performed the classification of different pests using FL on data obtained from UAVs, which are useful for crop applications due to their data collection flexibility and high spatial resolution.

Friha et al. [58] proposed a FL intrusion detection system, FELIDS, for securing agricultural IoT infrastructures. FELIDS uses deep neural networks, convolutional neural networks, and recurrent neural networks.

In [59], an Amendable Multi-Function Sensor Control (AMFSC) method for reducing the frequency of sensing and actuation is proposed. The method uses FL decisions by classifying the minimum and maximum production to modify sensor control. The control uses soil and sensed information.

In [60], a joint FL framework for Edge-assisted Internet of Agriculture Things (Edge-IoAT) is proposed to cope with both vertically and horizontally partitioned crop data in FL. The authors point out that excessive energy consumption may interrupt model training or prevent some edge nodes from communicating with the server, and inappropriate device scheduling may degrade model accuracy. In Edge-IoAT, energy and communication resources are limited since farm edge nodes, such as agricultural drones and iPads, are battery-powered. Due to limited spectrum resources, only some farm edge nodes can communicate with the server in each iteration. To solve this problem, Edge-IoAT uses energy-aware device scheduling to assign communication resources to the optimal subset of edge nodes.

In [61], the federated averaging model has been used to carry out crop classification using climatic parameters (temperature, humidity, pH, and rainfall) as independent variables and crop types (rice, maize, and chickpea) as labels. The model using the Adam optimizer converged faster than the Stochastic gradient descent (SGD) optimizer. The experiment with the farm dataset proved that the decentralized models perform better with faster convergence and higher accuracy than the centralized network models.

In [62], a multiple pest detection technique based on FL is presented and the original FedAvg algorithm is improved by adding a restriction term to prevent the local model from differing too much from the global model and to ensure convergence.

**Figure 2:**
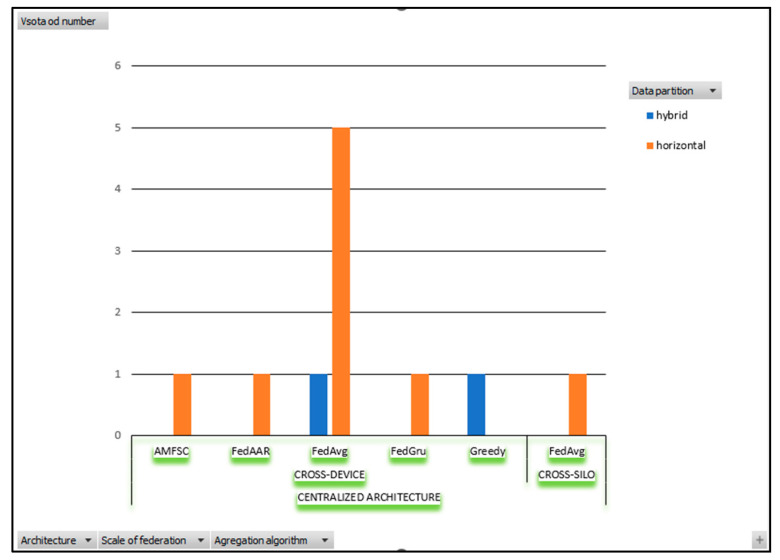
The architectures, the levels of federation, the data partitions, and the used aggregation algorithms for all considered FL systems in agriculture.

The architectures, the levels of the federation, the data partitions, and the aggregation algorithms used for all considered FL systems in agriculture are shown in Figure 2. All considered FL applications in agriculture have centralized architecture. One has a cross-silo level of federation with horizontal data partitioning, others have a cross-device level with horizontal, and two have hybrid data partitioning. FedAvg is the most often used algorithm for cross-device and cross-silo-considered FL applications in agriculture. 

### 4.2. Challenges of Production FL Systems

All considered FL systems in agriculture shown in Table 3 have small number of clients, but production FL systems can involve millions of devices in one netwoek. Such production FL systems faces a few challenges [55]: cost communication, heterogeneity, statistical heterogeneity, and privacy concerns. New challenges are productionizing and benchmarking in federated contexts are described ina [63].

The transfer of messages becomes slow due to low bandwidth, lack of resources, or geographical location in FL systems, which can involve millions of devices in one network.

Problems of production FL systems are [20]:

- Communication bandwidth: To provide efficient communication, the size of a message can be reduced using model compression schemes, and the total number of message transfers can be reduced;
- Privacy and data protection are concerns with FL, not about the local data that stays on the user’s device but about revealing the information from the model updates shared in the network;
- System heterogeneity with a large number of devices with differences in storage, communication, and computational capabilities, which cannot participate all the time. System heterogeneity can be managed with asynchronous communication, active device sampling, and fault tolerance;
- Statistical heterogeneity in FL systems is caused by data that are non-IID (not identically and independently distributed), with multiple variations of data, and with different precision or different-resolution images contained in the client devices.

In FL, edge devices connected over the network in a distributed environment must share their updates, and the high cost of communicating gradients can be a major problem in FL systems because the bandwidth of the participating user devices is limited. With privacy-preserving collaborative learning, FL increases communication costs with the continuous transfer of the large number of parameters (weights and biases) used by deep learning models between clients and the server. IoT and edge devices involved in FL have limited communication capabilities. A huge number of parameters and limited communication capabilities of devices form a communication bottleneck in FL.

#### 4.2.1. Communication Challenge of Production FL Systems in General and in Agriculture 

Communication costs become a greater problem of FL systems when the number of contributing clients and communication rounds increase. Communication is a critical bottleneck in FL, where data remain local, and the prediction model is sent between a potentially massive number of devices in the federated network [59]. But, a larger data set is required for FL to create a more robust and accurate model with a higher prediction accuracy since data from a small number of clients with small datasets usually limit the overall knowledge and performance of the ML model. 

The efficiency of communication between the central server and the clients can be improved in two ways: by reducing the number of communications and by reducing the size of transmitted messages. Proposed methods for reducing communication overhead in FL reduce the size of data with quantization or sparsification. In the quantization methods for reducing communication overhead in FL, high-precision parameters are quantized into low-precision values, while sparsification methods improve communication efficiency in FL by discarding redundant parameters. 

The second group of proposed methods for reducing communication overhead is censoring methods, which reduce all data of some participating devices [64]. Censoring methods adjust the communication frequency of each node regarding updates of the whole model after each round of the training process. In [65], a censoring method named the Communication-Censored Distributed Stochastic Gradient Descent Method-CSGD is proposed. In CSGD, at the beginning of each iteration *k*, the server sends its variable and threshold to all clients. Each client locally computes an estimate of its gradient, and then each client uploads a new gradient only when the distance between the calculated gradient at the client and the latest uploaded gradient before iteration *k* starts is greater than the censoring threshold. When the latest gradient is not available, the old is reused at the server. At the end of iteration k, the server only receives the latest uploaded gradients and updates its variable and the censoring threshold.

In agriculture, there is usually a huge number of devices with limited communication included in FL. The communication cost of sending local models through a computer network could be high because some networks, like ResNet, have more than 25 million parameters. 

End-user network connections operate at lower rates than the network connections that are available at a data center. Cross-device units in FL systems in agriculture usually use Wi-Fi or slower connections. The communication challenge is important in cross-device FL in agriculture because of many IoT devices or mobile devices [20]. Compression schemes like quantization and sparsification, which can be integrated with the aggregation algorithms, reduce the overall communication cost.

##### Sparsification Methods

Sparsification is a compression technique used in FL to compress the model for communication between server and client. Sparsification constructs sparse models from dense networks with lower storage and bandwidth requirements.

A Sparse Ternary Compression (STC) method provides compression for both upstream and downstream communications [66]. Sparsification compression is performed separately for the server model to address downstream communication and the client models to address upstream communication. It reduces the per-round communication cost of FL without significantly degrading the accuracy of the global model. STC extends the existing top-k gradient sparsification compression technique with a novel mechanism to enable downstream compression for efficient communication from server to client. The caching mechanism keeps the clients synchronized in case of partial client participation. Employing quantization and optimal lossless coding of the weight updates increases the efficiency. The SCT method is also highly effective when the communication bandwidth is constrained. 

FetchSGD [67] reduces the communication required in each round compared to many methods, which decreases the total number of communication rounds required to learn. In each round, clients first compute a gradient based on their local data, then compress the gradient using a data structure Count Sketch before sending it to the server. A Count Sketch is a randomized data structure that can compress a vector by randomly projecting it several times to lower dimensional spaces, allowing later recovery approximately. 

Gradient sparsification updates significant gradients and accumulates insignificant gradients locally. The accuracy after high sparsification can be preserved using the General Gradient Sparsification (GGS) framework, which corrects the sparse gradient updates with gradient correction and batch normalization updates with local gradients [68]. Gradient correction enables models to converge better since it works with the accumulated insignificant gradients. Batch normalization updates with local gradients can mitigate the impact of delayed gradients without increasing communication. Experiments performed on some neural networks like CifarNet and AlexNet proved that gradient sparsification reduces the communication overhead. 

##### Quantization Methods 

Quantization is a compression method for reducing the model size with a small decrease in model accuracy where the vector of weights and gradients is written in fewer bits, and the resolution is reduced. 

Reisizadeh et al. [69] proposed the FedPAQ FL method with periodic averaging and quantization. FedPAQ models are updated locally at devices and only periodically averaged into a global model at a server. Edge nodes quantize their updates before uploading them to the server. Furthermore, partial node participation enables the random availability of the edge nodes. 

Using the Lossy FL (LFL) algorithm, both the global and the local model updates are quantized before being transmitted [70]. The proposed LFL scheme quantifies the global model update rather than the global model itself. 

The Hierarchical Quantized Federated Learning (Heir-Local-QSGD) technique provides client–edge–cloud network hierarchy and quantized model updates [71]. It performs partial edge aggregation and quantization on the model updates. 

Mills et al. [47] proposed a communication-efficient FedAvg, called CE-FedAvg, which reduces the required rounds to achieve the desired accuracy and the total amount of data downloaded per round relative to FedAvg. 

Federated averaging with compression (FedCOM) was proposed for homogeneous datasets in [72]. It is a generalized version of the local stochastic gradient descent (SGD) method for FL that uses compressed signals to reduce communication overhead. FedCOM uses compressed messages for uplink communication. The new global model at the server combines the previous global model and the average of updated local models. These are the two differences between FedCOM and standard local SGD methods. A federated averaging with compression and a local gradient tracking algorithm called FedCOMGATE is very efficient for heterogeneous datasets. 

An online Model Compression (OMC) framework was proposed in [73], which stores model parameters in a compressed format and decompresses them when needed. Because the compression of parameters in OMC reduces model size, the communication cost caused by transporting models between clients and the server is reduced. Compression and decompression do not significantly slow down the learning process. OMC applies a linear transformation on the decompressed parameters to minimize the quantization error. OMC quantization uses per-variable transformation, weight matrices-only quantization, and partial parameter quantization. 

In [74], FedZip decreases the size of updates in the process of transferring weights from deep learning models between clients and their servers with only insignificant effects on accuracy or convergence speed. FedZip implements sparsification based on Top-k pruning and quantization with k-means clustering on the model’s weights. It encodes using Huffman encoding and two other encoding methods. FedZip offers a high compression rate with lower accuracy degradation. 

#### 4.2.2. Application of Aggregation Algorithms 

Aggregation algorithms for FL play a crucial role in the success of FL systems, as they integrate the knowledge of the participants. Many algorithms have been proposed since the first average aggregation algorithm proposed by Google FedAvg, which has several challenges in terms of performance [75]: convergence, high communication and computation cost, sensitivity to local models, different distributed data across the network, heterogeneity of devices, users and network channels, tuning difficulty, different characteristics of clients, and scalability problems. 

Some FL aggregation algorithms are described in Section 3.3., while several FL aggregation strategies and algorithms are explored in [24]. For efficient FL, practitioners must select the most appropriate method for their FL applications, and surveys (such as in ref. [76]) can help. The aggregation techniques have different emphases and purposes, such as enhancing performance, improving data security and privacy, or minimizing communication overhead. The survey [76]) divides aggregation techniques into four classes:

- Synchronous, where model aggregation occurs after all client updates have reached the server (like FedAvg);
- Asynchronous to handle device heterogeneity;
- Hierarchical to handle the presence of a large number of edge devices, such as IoT devices, using an edge layer to partially aggregate local models from closely related client devices before further aggregation;
- Robust aggregation with the purpose of ensuring secure aggregation throughout the FL process using encryption techniques.

## 5. Discussion

The use of spatial, temporal, and image data can provide improvements in prediction in agriculture. The FL is an appropriate machine learning technique because there are multiple data owners, and data privacy is important. 

We are aware that agriculture is a very broad concept. It can be divided into crop management with applications in disease detection, yield prediction, weed detection, crop quality, species recognition, and livestock management, including livestock production and animal welfare monitoring. Important areas also include water and soil management. In addition, there are many other agricultural-related research areas, including market, regulation, and intrusion detection systems for securing agricultural IoT infrastructures. 

We discuss the various FL applications and frameworks in many mentioned agriculture areas (see Table 3). 

We can conclude that the architecture of FL applications in agriculture is centralized. Most FL applications have a cross-device federation level, and only one FL application from Table 3 has a cross-silo level of federation with horizontal data partitioning.

The majority of considered FL applications in agriculture (see Table 3) have horizontal data partitioning and two of them have hybrid data partitioning. 

In considered FL applications in agriculture in Table 3, FedAvg is the most often used algorithm for cross-device and cross-silo FL applications and no censoring, sparcification or quantization method was used.

FL is a promising technique for a wide range of agriculture applications, including crop yield prediction, pest and disease detection, and precision farming. However, some challenges still need to be addressed before FL can be widely adopted in agriculture, such as the need for more efficient and secure aggregation algorithms and the use of model compression or sparsification techniques to solve the communication bottleneck of the FL systems.

The challenges beyond the technical issues to remove the bottleneck of FL and provide the use of FL in the agriculture sector at scale are further discussed. FL faces privacy issues, and a well-designed incentive mechanism is needed to encourage data owners to participate in FL systems. In executing the FL tasks, the data owners contribute their computation and communication resources. Data owners must protect their privacy because attackers may infer sensitive information from the updates sent to a server or recover the raw data based on the shared updates. Some practical incentive mechanisms for incentivizing data owners to join FL in the IoT area have been proposed. An example of such a mechanism is the DPFL framework, which jointly considers the data owners’ task expenditure and privacy risk [77].

In the ref. [78], the organizational challenge of FL applications with ample interdisciplinary opportunities, adoption strategies, and conceptual framework for the adoption of FL aplications strategies are discussed.

## 6. Conclusions 

FL is a new computing paradigm. In this study, we present FL and its applications in agriculture. We present the concept of FL, different architectures, scales of federation, and global model generation algorithms. Then, we analyze the applications of FL in agriculture. We discuss various proposed sparsification and quantization methods that can be used to overcome communication bottlenecks and enable FL to be widely adopted in agriculture. 

This survey helps researchers with the use of FL techniques and provides useful references in the field of agriculture. 

In future work, we will add the latest research advances in FL and plan to develop an FL benchmarking tool. 

## Figures and Tables

**Table 1:** Abbreviations for some DL algorithms/models/network types.

Abbreviation	DL Algorithms/Models/Network Types
CNN	Convolution Neural Network
RNN	Recurrent Neural Network
DNN	Deep Neural Network
ResNet	Residual Network
DBN	Deep Belief Network
DCNN	Deep Convolution Neural Network
MCNN	Multilayer Convolution Neural Network
DRL	Deep Reinforcement Learning
DenseNet	Densely Connected Convolutional Network
SGD	Stochastic Gradient Descent
MLNN	Multilayer Neural Network
GRU	Gated Recurrent Units
AlexNet	AlexNet Neural Network
SqueezeNet	SqueezeNe Deep Neural Network for Computer Vision
VGG-11	Very Deep Convolutional Networks for Large-Scale Image Recognition
ShuffleNet	ShuffleNet is a convolutional neural network designed especially for mobile devices with very limited computing power.
CMI-Net	Cross-Modality Interaction Network

**Table 2:** Federate learning architecture.

Type of FL	Data Partitioning	Sample Space	Feature Space	Use Case: Two or More Clients Share Datasets with:
Horizontal FL	Horizontal	Different	Same	The same feature space and different sample spaces make the dataset larger.
Vertical FL	Vertical	Same	Different	The same sample space and different feature space make the information about samples richer, which helps to build a more accurate model.
Federated transfer learning	Hybrid	Partial common sample space	Partial common feature space	Small common sample space and different feature spaces.

**Table 3:** Use cases of federate learning in agriculture.

Ref.	Agri Area	Number of Clients	Problem	Data Used	Challenges	FL Data Partition Method, Architecture	Aggregation Algorithms	Trained Model
[52]	Crop yield estimation.	3	Crop yield prediction.	Soybean yield dataset: weather, soil components, and crop data.	Data ownership, privacy preservation.	Horizontal FL, centralized architecture.	FedAvg	ResNet-based regression models such as ResNet-16 and ResNet-28.
[53]	Deep Privacy-Encoding-Based FL Framework for Smart Agriculture.	2	Intrusion detection.	ToN-IoT dataset.	Minimizing the risk of security and data privacy violation.	Horizontal FL, centralized architecture. FL server and edge devices such as a gateway/router connected to a large number of IoT devices.	FedGRU	GRU
[54]	The role of cross-silo FL in facilitating data sharing.	5	Facilitating data sharing across the supply chain in the agri-food sector.	Datasets for crop yield prediction from both imaging (remote sensing) and tabular (weather and soil data).	Data privacy.	Horizontal, central server-based FL.	FedBN (FLon non-iid features- via local batch normalization), extends FedAvg.	CNN and RNN.
[55]	Diagnosis of diseases in food crops.	4	Leaf disease prediction.	PlantVillage	Data privacy	Horizontal FL, centralized architecture.	FedAvg	Five CNNs: AlexNet, SqueezeNet, ResNet-18, VGG-11, ShuffleNet.
[56]	Automated animal activity recognition based on distributed data in the context of data heterogeneity.	5, 10 15 20 25 30	Automated animal activity recognition (AAR).	A public centralized dataset comprising 87,621 two-second samples that were collected from six horses with neck-attached IMUs.	Data Privacy.	Horizontal FL, centralized architecture.	FedAAR with gradient-refinement-based aggregation.	CMI-Net
[57]	EfficientNet deep model classifying nine types of pests.	4	Diagnoses of plant diseases.	Sensor technologies and IoT platforms, in conjunction with unmanned aerial vehicles (UAVs). The pest images.	Low computation power during the classification of pests for the agricultural environment.	Horizontal FL, centralized architecture.	FedAvg	Dense convolutional neural network (CNN) model combines pre-trained EfficientNetB3 with dense layers.
[58]	Intrusion detection system for securing agricultural-IoT infrastructures.	5, 10 15	Securing agricultural-IoT infrastructures.	Real-world traffic datasets -CSE-CIC-IDS2018, MQTTset, and InSDN.	Securing agricultural IoT infrastructures protects data privacy.	Hybrid data partitioning, centralized architecture.	FedAvg	Classifier: DNN, CNN, and RNN
[59]	Amendable Multi-Function Control Method using FL for Smart Sensors in Agricultural Production Improvements.	47	Improving productivity.	Crop and soil data.	FL from sensing data.	Horizontal	Amendable Multi-Function Sensor Control Method (AMFSC).	AMFSC
[60]	Agricultural production.	10	Guiding agricultural production.	The images of real-world soybean iron deficiency chlorosis (IDC)dataset.	Fast convergence rate, low communication cost, and high modeling accuracy under resource constraints	Hybrid data partitioning, centralized architecture.	Proposed a joint FL framework for Edge-assisted Internet of Agriculture Things (Edge- IoAT) framework and a greedy algorithm to find the optimal solution.	Greedy algorithm,
[61]	Crop classification, smart farming	6	Data privacy in smart farming	The dataset with independent variables of temperature, humidity, pH, and rain	The application of FL to smart farming	Horizontal FL, centralized architecture.	Federated averaging model	CNN
[62]	Multiple diseases and pest detection	6	To avoid high data storage and communication costs, an unbalanced and insufficient data from orchards, diversity of pests, and diseases, and complex detection environments by traditional cloud-based deep learning.	Images, 445 orchard apple pictures, of which only 152 pictures contain 5 diseases	To solve the problem of unbalanced and insufficient data, avoid the communication cost generated by a large amount of data upload	Horizontal FL, centralized architecture.	FedAvg	Improved faster region convolutional neural network (R-CNN)
